# A Stochastic Model of DNA Fragments Rejoining

**DOI:** 10.1371/journal.pone.0044293

**Published:** 2012-09-13

**Authors:** Yongfeng Li, Hong Qian, Ya Wang, Francis A. Cucinotta

**Affiliations:** 1 Division of Space Life Sciences, Universities Space Research Association, Houston, Texas, United States of America; 2 Department of Applied Mathematics, University of Washington, Seattle, Washington, United States of America; 3 Department of Radiation Oncology, Emory University, Atlanta, Georgia, United States of America; 4 NASA, Lyndon B. Johnson Space Center, Houston, Texas, United States of America; National Taiwan University, Taiwan

## Abstract

When cells are exposed to ionizing radiation, DNA damages in the form of single strand breaks (SSBs), double strand breaks (DSBs), base damage or their combinations are frequent events. It is known that the complexity and severity of DNA damage depends on the quality of radiation, and the microscopic dose deposited in small segments of DNA, which is often related to the linear transfer energy (LET) of the radiation. Experimental studies have suggested that under the same dose, high LET radiation induces more small DNA fragments than low-LET radiation, which affects Ku efficiently binding with DNA end and might be a main reason for high-LET radiation induced RBE [1] since DNA DSB is a major cause for radiation-induced cell death. In this work, we proposed a mathematical model of DNA fragments rejoining according to non-homologous end joining (NHEJ) mechanism. By conducting Gillespie's stochastic simulation, we found several factors that impact the efficiency of DNA fragments rejoining. Our results demonstrated that aberrant DNA damage repair can result predominantly from the occurrence of a spatial distribution of DSBs leading to short DNA fragments. Because of the low efficiency that short DNA fragments recruit repair protein and release the protein residue after fragments rejoining, Ku-dependent NHEJ is significantly interfered with short fragments. Overall, our work suggests that inhibiting the Ku-dependent NHEJ may significantly contribute to the increased efficiency for cell death and mutation observed for high LET radiation.

## Introduction

Exposure to high linear energy transfer (LET) radiation occurs from radon gas in homes [2], the neutron components of doses to survivors of the Atomic-bombs in Japan or reactor workers [3], secondary neutrons in high altitude aviation [3], protons and carbon beams that cancer patients are exposed to [4], and cosmic rays doses to astronauts during space travel [5], [6]. High LET radiation has been shown to have increased effectiveness for mutation and cancer and is attributed to the complexity of DNA damage produced in comparison to low LET radiation such as X-rays or 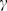-rays. However, the mechanism for the increased effectiveness observed for high LET has not been fully elucidated. DNA double-strand breaks (DSBs) are induced by ionizing radiation (IR) and other agents, leading to a large number of DNA damage response signals in a cell, and are responsible for cell lethality, mutations and genome instability. Homologous recombination repair (HRR) and non-homologous end joining (NHEJ) are the main pathways used to repair DNA DSB in mammalian cells. There are two types of NHEJ repair pathways, nominal NHEJ which is Ku-dependent and back-up NHEJ denoted as B-NHEJ, which is Ku-independent. Ku protein is a heterodimer consisting of Ku70 and Ku80. In the Ku-dependent NHEJ [7], [8], once DSBs are formed after the exposure to IR, DNA free ends will first recruit Ku and subsequently other DNA repair proteins, including the catalytic subunit of DNA-dependent protein kinase (DNA-PKcs), XRCC4/Ligase IV, XLF and so on, for DNA end processing and ligation. In contrast, the Ku-independent B-NHEJ is a slow but efficient alternative repair pathway in the absence of Ku and requires other proteins such as PARP1 [9], [10]. Clustered DNA lesions, consisting of DSBs, single strand breaks (SSBs) and base damage, are more difficult to repair and even irreparable, which are often attributed to the high efficiency for cell death, mutation and cancer [2]–[6], [11]–[13], however the details on the mechanism are lacking.

Besides experimental studies, mathematical modeling has drawn much attention of biologists and provided an alternative tool to investigate DNA repair. Because DNA repair involves a large biochemical network, a model usually leads to a large system requiring numerical simulation, or reduces into a simple form by ignoring some details so that the qualitative features become accessible by analytical treatment and can still provide valuable insight into the system. DNA repair has been studied by mathematical modeling in some works. The importance of formation of an enzyme-substrate complex in rejoining was described in [14]. Some models were based on the Ku-dependent NHEJ pathway through Monte Carlo simulation [15] or mathematical analysis [16], [17], or HRR pathway [18]. Additionally, other models focused on the DSB rejoining kinetics [19], [20] or the kinetics of the damage and repair probability [21].

In this work, we proposed a mathematical model of DSB repair through DNA fragments rejoining according to the simplified Ku-dependent NHEJ mechanism, which focuses on the Ku binding step and suppresses the details of later steps in NHEJ, in order to highlight the rejoining kinetics for different fragment lengths. The subsequent steps in NHEJ were treated in our previous work [16], [17] and can be added to the model at a later time. It is believed that the binding efficiency of two Ku heterodimers to the two ends of a DNA fragment at the same time is determined by the length of the fragment [22], [23]. It has been shown recently by experiments [1] that at the same dose, high LET radiation produces more short DNA fragments (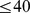 bp) than low LET radiation. Thus we introduce two quantities in the model: a minimum length 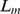 and a critical length 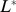. Precisely one DNA fragment of length 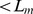 cannot recruit a Ku protein and hence such small fragments cannot be rejoined with others and supposedly will be discarded under degradation or perhaps repaired through an alternate pathway. Conversely one DNA fragment of length 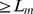 can recruit one Ku molecule on one end, but not two Ku molecules on two ends simultaneously unless its length is larger than the critical length 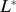.

For a prescribed initial fragment distribution, by Gillespie's direct method of stochastic simulation [24], we are able to mimic the random kinetics of the DNA fragments rejoining and estimate rejoining time, the time spent on a complete fragments rejoining process. We will show that the effects of various factors on the repair of DSB are described from a mechanistic basis, that can explain the increased biological effectiveness of high LET radiation.

## Model

**Figure 1:**
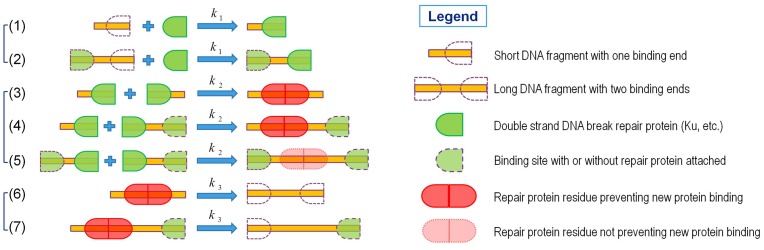
Model of DNA Fragments Rejoining. Here 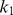, 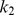 and 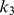 are the recruitment rate of repair protein, the joining rate of fragments and the release rate of repair protein residue, respectively. A short DNA fragment of length 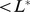 has only one binding site (1) and a long DNA fragment of length 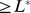 has two binding sites (2). Joining with a short DNA fragment results in the formation of a protein residue that prevents the recruitment of new protein on both ends (3) or on only one end (4). The residue resulting from the joining of two long fragments is ignorable because it does not affect the recruitment of new proteins (5). Non-ignorable protein residue must be released to make binding site(s) accessible by new proteins(6–7).

According to the Ku-dependent NHEJ pathway, we proposed a mathematical model of simple DNA fragments rejoining to study DNA repair and how it is affected by radiation quality where increases in smaller fragments are observed for high LET radiation. This model of DNA fragments rejoining consists of three steps, recruitment of repair protein, rejoining of fragments and release of repair protein residue, which are illustrated in Fig. 1 and will be discussed shortly. We assume that all of the three steps of reactions are irreversible. Because NHEJ is expected to be error-prone, we refer to completion of the mathematical process as rejoining rather than complete repair, since in many cases several base pairs may be lost at the location where fragments join, and also we will not consider the possibility of joining of fragments on distinct chromosomes or distant regions from the initial fragment on single chromosomes.

### Recruitment of Repair Protein

After DSBs are induced by ionizing radiation, Ku is the first protein that binds with the DNA free end. Ku is a heterodimer consisting of Ku70 and Ku80 that form a ring surrounding DNA. Once binding to the DNA free end, Ku translocates inward so that the free end is exposed for binding with other repair proteins such as DNA-PKcs, XRCC4/Ligase IV and XLF. In the following, we refer to Ku as the repair protein denoted by E, and suppress the description of the binding of other NHEJ proteins. Because Ku is abundant [25], in this model, we assume that the repair protein E is abundant and remains constant. Let 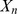 denote the DNA fragment of length 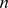, that is, 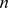 base pairs (bp).

Due to the binding to DNA and subsequent translocation of Ku, the DNA fragment must be long enough to hold Ku to form a stable complex. Experiments revealed that Ku translocates inward by approximately 14 bp on the DNA [26], but probably 20 bp is needed for efficient binding [23]. On the other hand, a longer DNA fragment can recruit more than one Ku heterodimer. By experimental study, one DNA fragment of 42 bp can bind with two Ku heterodimers at its two ends and a DNA of 75 bp or more can accommodate three dimers, two at the ends and one in the middle. Therefore it is reasonable to assume that there exist a minimum length 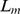 and a critical length 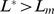, such that a DNA fragment 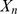 can recruit one repair protein E as 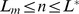 and recruit two as 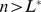. In this model, we set
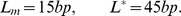
We could also speculate that, besides the one identified for the Ku protein, there are other critical lengths that would be related to the binding of DNA-PKcs and other repair proteins when clustered DNA damages occur. As seen shortly, our mathematical model would be amendable in a straight-forward fashion if larger critical lengths related to repair complexes are identified in the future.

As shown in Fig. 1(1–2), a short DNA fragment has only one end capable of binding with repair protein, but a long fragment has two ends available for binding. The recruitment of the repair protein can be written in terms of chemical reactions as:
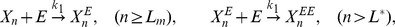
where 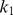 is the recruitment rate of repair protein, 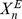 and 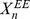 are DNA complex bound with one and two repair proteins, respectively. Indeed DNA binding with repair protein is a reversible process, the rates of binding and unbinding depend on the length of fragments. The study of [23] suggested that longer fragments yield more efficient binding with a repair protein. Moreover, Ku shows high affinity for and slow dissociation rate from DNA [27]. In this model, therefore, we assume that the recruitment is an irreversible process with a constant recruitment rate 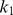 for all the fragments of length 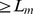. Note that the irreversible recruitment process is an approximation of the reversible one with small unbinding rate. This approximation may change the kinetic profile of fragments rejoining quantitatively, but not qualitatively, as discussed in Appendix S1 (Note 1).

### Fragments Rejoining

Once repair proteins are bound, two pieces of DNA fragments, 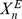 and 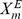, can join together at their ends and form a new single DNA fragment 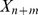 of longer length 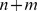. We assume that the fragments always undergo perfect rejoining, that is, no breakup after rejoining, thus this rejoining process is irreversible, and that any pair of fragments join at the same rate 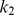. The resulting new fragments, however, may not be ready to recruit new proteins freely due to the repair protein residue that remains on the new fragment and may prevent the recruitment of a new protein. For example, if both fragments are short, that is, 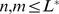, both ends of 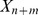 cannot recruit the new protein unless the protein residue is released for a clean DNA free end, as shown in Fig. 1(3). The reaction for this fragments rejoining is given by
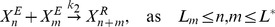
where R denotes one pair of repair protein residues preventing the recruitment of new proteins on both of two ends. While if among two fragments one is short and the other is long, that is, 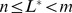, then the residue affects only the end of 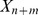 inheriting from 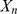, but not the one from 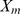, as shown in Fig. 1(4). The reactions for the fragments rejoining in this category are
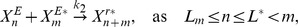
where 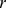 is one pair of repair protein residues preventing the recruitment of new proteins on only one end, 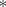 can be either none (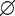) or protein E or residue r. Then 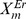 denotes a fragment with protein on one end and protein residue close to the other end, and 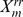 is a fragment with two pairs of protein residues with the first one being close to one end and the second one close to the other end. At last if both fragments are long, that is, 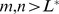, then the protein residue on the resulting fragment has no impact on the recruitment of new protein on either end and hence such residue can be ignored, as shown in Fig. 1(5). The reaction for such fragments joining is simply
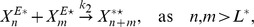
where 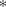 and 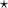 are 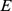, 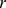 or 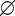.

### Release of Repair Protein Residue

As shown in Fig. 1(3–4), after fragments rejoining, the protein residue is so close to the binding site that it can prevent the recruitment of new protein. Therefore, the residues must be removed to release these sites for binding with the new proteins. This is the release step as shown in Fig. 1(6–7). It has been poorly understood for a long time how Ku is released from the DNA after the end joining, until recent work revealed that Ku is released with ubiquitin [27] or by Mre11 nuclease activity and Ctp1 [28]. In the model, we assume that the repair protein residue cannot bind to DNA after it is released, that is, the release process is irreversible, and that all of the release processes share the same release rate 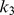. Then the reactions for the release of protein residue are given by
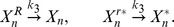
Furthermore, if the length of resulting fragment is larger than 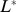, then it can recruit protein on both ends after the residue is released, see Fig. 1(6–7). However, if its length is less than 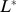, then it still has only one end capable of binding new protein, and it need bind with more fragments to reach the critical length. Recall from the previous section that a fragment generated by joining two long fragments (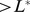) does not need a release step, which leads rapidly to complete rejoining. Therefore, the DNA rejoining time will rely heavily on the number of short fragments.

### Initial DNA Fragment Distribution

DNA fragments rejoining involves in three steps of reactions and leads to a large system of biochemical reactions, see Appendix S1 (Note 2). To investigate this model of fragments rejoining, an initial condition is needed to start the simulation of the model. Radiation, particularly high LET radiation, can produce short DNA fragments due to the clustering of damage close to a particle track [29], [30]. A random distribution of different fragment sizes occurs as dependent on the LET or radiation quality, and the dose. If the fragment is even shorter than 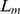, it will not be able to bind with Ku and hence cannot be repaired through the Ku-dependent NHEJ pathway. For convenience of numerical study of the fragments rejoining time, we assume that such short fragments will not undergo any rejoining by alternative pathways. In other words, only fragments eligible for binding Ku are counted in the initial fragment distribution, which, therefore, can be written as 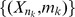 or 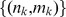, where 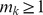 is the number of fragments of length 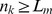. By omitting all these small fragments (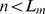) from the initial fragment distribution, the fragments rejoining is not complete due to the loss in the real situation. Nevertheless, the model itself describes a complete fragments rejoining in the sense that all fragments counted in the initial distribution are eventually joined to become one piece of DNA.

Nevertheless, short fragment (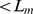) does matter in the biological effect of radiation because it is one of the main counts for the unrepaired DNA damage leading to cell death or mutation. Hence short fragments of 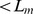 will be taken into account in the initial fragment distribution when comparison of numerical simulation with experimental data is needed.

## Results

### Stochastic Simulation and Mean Rejoining Time

Because 1 Gy radiation induces 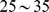 DSBs, a discrete model with only a few number of molecules fits more into the study of fragment rejoining than a continuous model of reaction rate equations by the mass action law. For a given positive integer 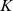, let 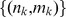 be a sequence of positive integer pairs such that 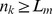 and 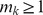 for 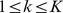. A prescribed initial DNA fragments distribution is written as
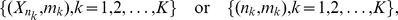
which consists of 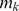 pieces of DNA fragments of length 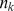. The total number, total length and mean length of the initial fragments distribution are given by
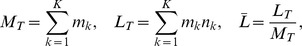
respectively. Starting with the initial fragments distribution, DNA fragments will undergo a series of irreversible processes, that is, recruit the rejoining proteins, join with one another and release a protein residue, until only one fragment is left when the rejoining is complete. The DNA rejoining time is defined as the total time span from the initial 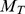 fragments decreasing to the final one fragment. As you will see shortly, both 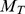 and 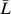 play crucial roles in the DNA rejoining time.

At the molecular level, all the involving reactions in the fragments rejoining are in the random manner, thus we will perform the stochastic simulation by Gillespie's algorithm [24] to mimic the kinetics of fragments rejoining. Besides all the parameters we have introduced, the volume 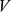 of chemical solution also matters by influencing the second order reactions such as protein recruitment and fragments joining. Since DNA is located in the nucleus, we take V to be the volume of the nucleus.

**Figure 2:**
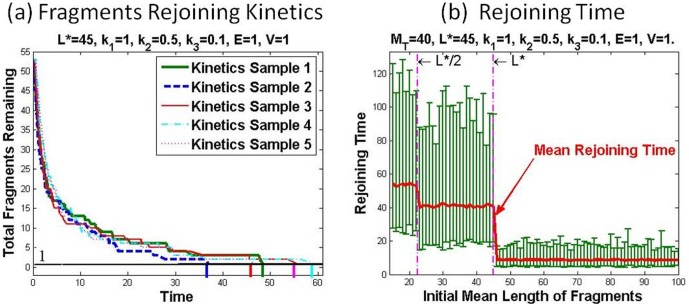
Stochastic Simulation of Fragments Rejoining. Panel (a) shows the sampled kinetics of stochastic fragments rejoining starting with the same initial fragments distribution. Panel (b) shows the fluctuations of rejoining times around their mean rejoining times against the mean length of initial fragments.

To illustrate the kinetics of fragments rejoining, we suppose that there are initially 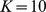 species (DNA fragments of different length), the fragment length 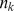 and amount 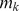 satisfy uniform distribution on the intervals 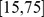 and 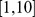, respectively, by which a randomly picked initial fragment distribution 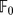 is given below and has totally 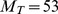 pieces of fragments with mean length 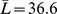 bp.
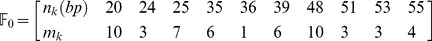
By Gillespie's direct method of stochastic simulation, for which more details can be found in Appendix S1 (Note 3), the kinetics of fragments rejoining is given in Fig. 2(a). The randomness in the fragments rejoining infers that the rejoining time can be very different even for the same initial distribution, as shown in Fig. 2(a). The fluctuation of rejoining time of different samples from their mean values is illustrated in Fig. 2(b) against the mean length. To calculate the mean rejoining time, we assume that all the initial fragments have the same length. In other words, suppose that initially 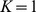 and 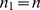, then 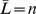. For each given n, specific amount (150) of samples are used to calculate the rejoining time and the mean value of rejoining times from all samples is taken as the mean rejoining time. The error bar shows the fluctuation of the rejoining time away from their mean value, the top and bottom of the bar is the maximum and minimum rejoining times, respectively, among the samples used for this calculation. It is readily observed that the rejoining time varies over a large range when the initial mean length is less than the critical length (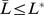), compared to that as 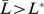. Intuitively as 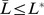, the fragments joining needs one more step, the release process, suggesting that more types of reactions increase the complexity by introducing more randomness of the reaction system.

### Impact Factors of the Mean Rejoining Time

By numerical analysis, our model can describe how the mean rejoining time is affected by numerous factors including the nuclear volume, release rate of repair proteins, and the initial fragment distribution.

**Figure 3:**
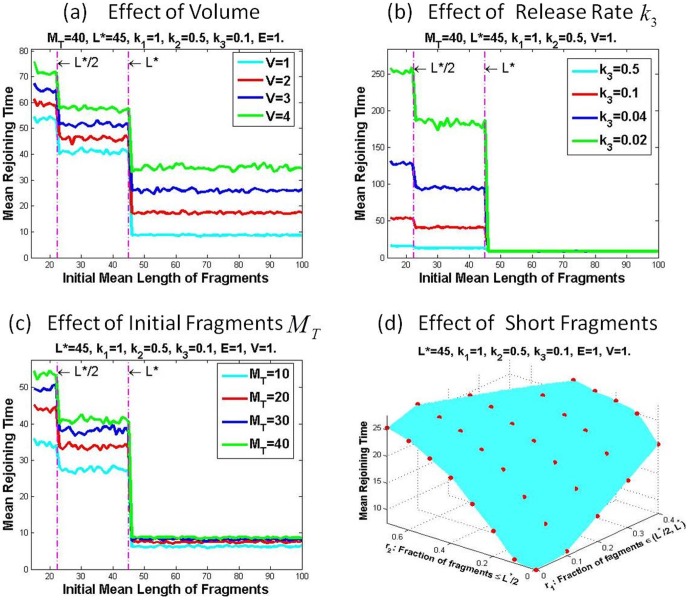
Impact Factors of the Mean Fragments Rejoining Time. The mean rejoining time is affected by different factors (a) volume of the nucleus, (b) release rate 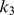, (c) initial number of fragments and (d) fraction of short fragments. (a)–(c) all show that more short fragments (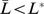) lead to longer rejoining time, which is summarized in (d), where red dots are the simulation data interpolated by the cyan surface.

In the chemical reactions, the high order reaction rate depends on the volume of the chemical solution. Because both repair protein recruitment and fragments rejoining are second order reactions, their efficiencies will depend on the volume of the nucleus where DNA is located. Since the nucleus varies in size at the different stages during the cell cycle, it is important to know how the nucleus volume affects the DNA fragments joining. As shown in Fig. 3(a), the larger the volume is, the longer the rejoining time. For the same type of cells, the size of nucleus increases when cell enters the G2/M phase from G1 phase. This verifies that cells are more sensitive to the radiation in the G2/M phase than that in the G1 phase. Conversely S-phase cell shows resistance to the low LET radiation even though its nucleus is enlarged, probably because HR repair takes place, in addition to the NHEJ, during the S phase.

After two pieces of DNA fragments join, the repair protein residue must be released before recruiting new repair protein if one piece of fragment is less than the critical length 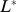. If all the DNA fragments have length larger than 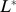, then the release step is not necessary and hence varying 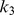 has no effect on the rejoining in this case, as shown in Fig. 3(b) for 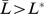. While if 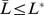, the release rate 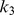 has significant impact on the rejoining time. Because smaller release rate requires longer release time, it eventually increases the rejoining time. Fig. 3(b) shows that the rejoining time is increased markedly as release rate is reduced by half when 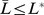.

It is believed that the number of radiation-induced DSBs is proportional to the radiation dose, varying from 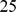 to 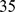 DSBs per Gy in different cell lines [31], regardless of low or high LET. Fig. 3(c) shows that the mean rejoining time is elevated with increased amount of fragments, suggesting that with the same initial mean length of fragments, more fragments result in longer rejoining time.

From Fig. 3(a–c), we observe that the mean rejoining time is almost the same for all values of mean length 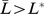. Because if all the fragments have lengths 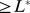, there is no release step of protein residue throughout the entire rejoining process; all the fragments recruit repair protein and join with one another with efficiency independent of the length. More precisely, with initial 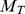 fragments, the entire rejoining process consists of 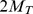 steps of protein recruitment (each fragment needs two proteins) and 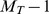 steps of fragments rejoining, leading to the same mean rejoining time regardless of the value of 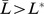. Furthermore, as shown in Fig. 3(a–c), the mean rejoining time exhibits a big jump at 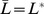. In comparison with 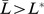, each rejoining among fragments 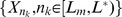 is followed by a release step to proceed the process in addition to all the reactions in the case of 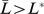. The extra time for release step results in longer rejoining time and accounts for the abrupt jump at 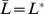. Interestingly, another jump occurs at 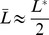. Decompose the length range into 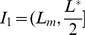, 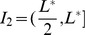 and 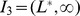, and consider two fragments 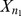 and 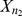 such that 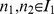 and 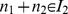. Then the resulting fragment 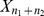 is longer but still needs to join with more fragments to form a longer one in the range 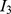, requiring more release steps. Consequently, by this discussion, we may expect a jump occurring at the mean length given at 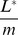 for any positive integer 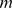, if fragment 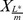 can recruit repair protein. Therefore, we conclude that with fixed initial amount, shorter DNA fragments demand more time for a complete rejoining.

In the real situation of DNA damage, the length of DNA fragments varies. Given an initial distribution with fixed amount of DNA fragments, let 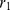 and 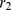 be the fractions of short DNA fragments of length in 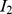 and 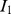, respectively, then 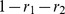 is the fraction of long fragments in 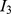. To study the effect of fraction 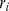 on the rejoining time, we randomly picked 5000 fragment distributions 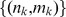 under the constraint that (i) 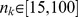
bp, (ii) 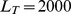 bp and 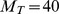. A total of 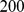 samples are used for the stochastic simulation to calculate the mean rejoining time associated to each fragment distribution that corresponds to a fraction pair 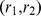. By 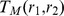 we denote the mean value of all the mean rejoining times associated to the same fraction pair 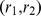, illustrated in Fig. 3(d). 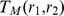 is increasing in both 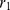 and 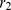, indicating that more short fragments lead to more time for complete rejoining and hence lower the rejoining efficiency, and consequently suggesting an important contribution to increased cell death. The result is consistent with the experimental observation that high LET radiation inhibits Ku-dependent NHEJ pathway, but not HR and PARP1-dependent NHEJ [1]. It also explains that S-phase cells become more sensitive to high LET radiation, while resistant to the low LET radiation at the same dose because high LET IR produces more short fragments than low LET IR of the same dose [32].

### Comparison with Experimental Data

In the above discussion, all the simulations are for the initial fragments distribution without counting the fragments shorter than the minimum length 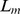. In the experiments, there are always some DNA DSBs that are not repaired. In this section, we will take into account these extremely short fragments that cannot recruit Ku protein for subsequent NHEJ repair pathway and hence count for the irreparable DSBs, which result in unrejoined DSBs and likely increase the probability of mis-rejoining leading to chromosomal aberrations and other mutations. These unrejoined breaks will appear as long lasting foci observed in the experiments and suggest the cells resistance to the DSB repair.

Because 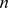 DNA DSBs result in 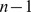 DNA fragments (if two fragments on the two ends of the DNA sequence are not counted), the amount of radiation-induced DSBs reflects the amount of produced DNA fragments. In several papers [12], [33], the repair kinetics of DNA DSBs was discussed by studying the change of 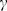-H2AX or 53BP1 foci, as a marker of DSB. Computational models have been used to study the distribution of large fragments (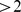 kbp) [34], and in some cases to the probability of two DSBs within the 160 bp nucleosome [35] which will lead to short fragments. Estimates for low LET radiation such as X-rays or electrons suggest that the probability that a DSB occurs with the addition of a second DSB within the same nucleosome is less than 5% [35], however, for high LET radiation such as 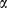 particles or heavy ions, this probability can exceed 30%. Calculations of the distribution of small fragments sizes have not been reported, however we estimate that a large fraction of the interactions leading to two or more DSBs within the same nucleosome will lead to fragments below the critical length of 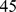 bp.

Experiments measuring DNA repair foci as a marker of DSB repair lead to biphasic kinetics [13], [31], [33]. Our kinetic model naturally leads to such a biphasic description where long DNA fragments (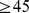 bp) are joined through fast kinetics and short DNA fragments (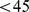 bp) are joined through slow kinetics. We compared results of our mathematical model to foci experiments [13] using estimates from previous reports [20], [30], [35] that DNA damage induced by high LET radiation, such as 1 Gy Fe ion, consists of 70% long DNA fragments and 30% short fragments, while a much lower proportion of short fragments is produced by low LET 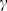-rays, 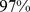 and 3%, respectively. It is not clear about the exact percentage of short fragments produced or their initial structures that are directly generated by high-LET Fe ion in cells. Fe ions have large energy deposition in what is known as the track core close to the particles track, and small energy deposition in the track penumbra due to the secondary electrons ejected by the particle as described in [29], [30], [34], [35]. Rydberg et al. were able to show an enhancement in small fragments for heavy particles such as Fe ions [30], [40]. However they could not resolve the smallest fragments below a few hundred base pairs. Nevertheless theoretical models suggest that in the track core, 30% or more DSBs will be produced close together at high LET as was predicted by Nikjoo et al. [35] and earlier work cited in this paper. Furthermore, as previously reported [36], [37] that, dual cutting of closely opposed AP/abasic sites (within 6 bp) generates so called *de novo* DSBs, which account for 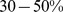 of total radiation-induced DSB and are major contributors to cytotoxicity. Since high-LET radiation generates more clustered DNA damage than low-LET radiation, it is reasoned that high-LET irradiated cells should have more such small DNA fragments than low-LET irradiated cells. Although no exact yield of the short DNA fragments in cellular DNA has been reported since such small fragments are very difficult to be directly detected and counted for the quantity, we have reported that compared with the low-LET irradiated cells, the high-LET irradiated cells showed 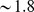 fold of such small fragments at the initial DNA damage directly generated by radiation [1].

**Figure 4:**
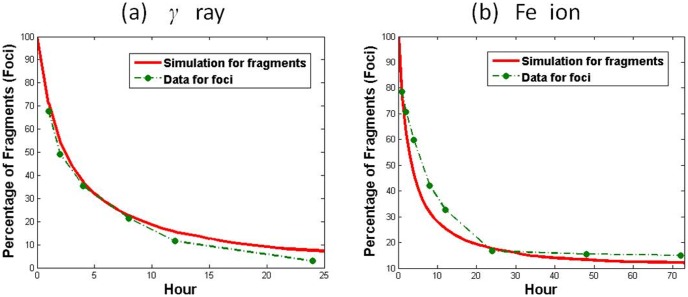
Mean Kinetics of DNA Fragments Rejoining Induced by Low and High LET Radiation. According to [13], [20], [30], [35], we assume that 1 Gy Fe ion produces 70% long fragments and 30% short fragments, while 1 Gy 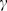-ray produces 97% long fragments and 3% short fragments. The red solid line is the numerical simulation of the mean kinetic change of the percentage of remaining fragments 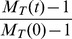, in comparison with foci data [13] indicated by the green dot-dashed line.

To compare with experimental data, parameters are properly chosen and simulation results for fragments are adjusted to be comparable with foci data, see Appendix S1 (Note 4). The mean kinetics of DNA fragments rejoining is obtained from the average of totally 200 samples of stochastic simulations by Gillespie's algorithm. As shown in Fig. 4, the kinetics of fragments rejoining in the decreasing manner indicates that cells undergo continuous DNA repair by rejoining all the repairable fragments, which is consistent with foci experiments [13].

We have not considered the role of complex DSBs resulting in a long fragment but slower repair due to additional end processing steps [33], which were described in earlier models [16], [17]. Also, the model counts all the fragments in the simulation, but the amount of foci observed in the experiments does not accurately point to the number of breaks. It is shown in [13] that the 53BP1 foci are different in size. It is very likely that a large focus consists of numerous small foci, each of which is for one DSB but not distinguishable experimentally. Both experiments and numerical simulation show the existence of unrepaired DNA lesion, or isolated DNA fragments that are not rejoined with others. It is believed that the irreparable clustered DNA lesion is due to the complexity of the damage. Our mathematical study suggests, from a different angle, that the unrepaired DNA damage may be due to the relatively large amount of irreparable short DNA fragments produced frequently by high LET radiation compared to those produced by low LET radiation.

## Discussion

Because unrepaired DSBs can lead to the cell death, the functioning of NHEJ, especially the Ku-dependent NHEJ, repair pathway is closely related to the cell radiosensitivity or cell survival. This consists of several aspects. First, the repair efficiency is related to the initial amount of DSBs [38], especially if the number of available repair proteins is limited or the presence of nearby DSBs affects the repair of any particular one. Second, the DSB rejoining rate affects the repair efficiency [39]. Third, the efficiency of DSB repair also depends on the severity of the damage, including both the quality of DSB and the spatial distribution of DSBs. It is known that under the same dose, high linear energy transfer (LET) IR causes more clusters of DSBs leading to short DNA fragments (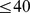 bp) [20], [30], [40], which are likely related to increased efficiency for cell death and mutation compared to low LET IR. Recent study also showed that high LET IR affects efficient Ku-dependent NHEJ but does not affect Ku-independent B-NHEJ [1]. This further highlights the important role of Ku-dependent pathway in the DSB repair when the different effects of high and low LET IR on the cell damage and survival are pursued. Although the structure of DNA end may also play an important role in the DSB repair by affecting the fidelity of DSB rejoining, experiments revealed that the repair efficiency is similar for most types of DNA ends produced by low LET radiation [41], while less is known for high LET radiation. Cells mutated for prominent repair proteins important for processing of complex DSBs such as DNA-PKcs, ATM, and NBS are shown to have a reduced role in the formation of chromosomal aberrations for high LET radiation compared to low LET radiation [42]. Thus the spatial distribution of DSBs leading to inhibited Ku binding significantly contributes to the increased effectiveness of high LET radiation.

In the paper, we proposed a mathematical model for DNA repair through simple DNA fragments rejoining. Some critical lengths were introduced to distinguish the fragments that are able or unable to bind with one or two Ku heterodimers simultaneously with one on each of their two ends. Because high LET radiation such as heavy ions and 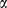 particles produces shorter DNA fragments, we were interested in how the fragment length alters the kinetics of DSB rejoining. By stochastic simulation, numerical results revealed that the mean rejoining time is affected by various factors, including the fragments rejoining rate, protein residue release rate, initial mean length of fragment, the critical length, and the initial amount of fragments. In the model, the critical lengths, 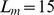 bp and 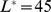 bp, were set according to Ku. We could also speculate that there are different critical lengths associated with other NHEJ proteins such as DNA-PKcs, for which our model would be easily amended once identified.

For a prescribed initial fragment distribution, by Gillespie's direct method of stochastic simulation [24], we are able to mimic the random kinetics of the DNA fragments rejoining and estimate rejoining times, the time spent for a complete fragments rejoining process. Numerical simulation showed significant differences between the rejoining times of DSB damage, with the initial fragment distribution whose mean length 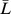 is either larger or smaller than the critical length 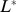, suggesting that, given initial amount of fragments, the rejoining time heavily relies on the mean length of initial fragments. This discovery is consistent with and provides a reasonable explanation for the fact that high LET IR induces more cell death and mutation than low LET IR of the same dose. As concluded in [1] high LET IR generates more short (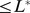) DNA fragments than low LET IR, although they may induce approximately the same initial number of DSBs. Therefore, more short fragments and consequently smaller mean lengths (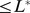) result in longer rejoining time, and thus increase the probability for mis-rejoining and deleterious biological effects. This is exactly as proposed in [43] that the binding features of Ku to DSBs determine the biological sensitivity to high LET IR. Overall, the model integrates the effects of various factors on the repair of DSBs caused by IR, especially for high LET IR, and provides a testable hypothesis to explain the increased biological effectiveness of high LET radiation.

It is important to know the initial fragment distribution, especially the fraction of short DNA fragments related to the end point of cell death. However, very few relevant experimental data has been reported because small fragments are hard to be detected, and the prediction of such distributions requires a different type of modeling approach related to Monte-Carlo simulation of radiation tracks and DNA structures and is beyond the scope of the present work. As far as concerned about the fraction of short fragments induced by Fe ion.

From theory, if a DNA DSB fragment at any length is not repaired the cell should die. In fact, although DNA DSB fragments 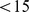 bp could not be repaired by NHEJ; however, if the 15 bp fragment does not locate in a critical gene, the cell will not die. We believe that our model will guide biologists to design their future experiments that will provide additional data to verify the model.

In addition, it should be noted that the repair enzymes not only play the role of DNA repair, but also cause cell death because the repair of two nearby DNA lesions may lead to the formation of a DSB. This double-edged sword effect of repair enzymes was considered in [44], suggesting an optimal level of the repair enzyme for balancing the repair and death. This important effect deserves consideration in our future work on this subject.

## Supporting Information

Appendix S1 Mathematical details related to the model development and numerical solutions to the model described.
(PDF)
