# Supplementary material for: A Stochastic Model of DNA Fragments Rejoining
Source: PLoS One. 2012 Sep 13;7(9):e44293. doi: 10.1371/journal.pone.0044293 (PMC3441539; doi:10.1371/journal.pone.0044293)
Supplement: Appendix S1 — Mathematical details related to the model development and numerical solutions to the model described. (PDF) [file pone.0044293.s001.pdf]

Appendix S1

Supplementary Note 1: Irreversible Approximation

Consider a reaction system consisting of a reversible reaction and an irreversible reaction,

$$x + y \underset{\beta}{\overset{\alpha}{\rightleftharpoons}} z, \qquad z + u \xrightarrow{\gamma} v. \tag{1}$$

It is known that system (1) eventually tends to the equilibrium state $x = z = 0$ no matter whether the first reaction is reversible ($\beta > 0$) or irreversible ($\beta = 0$), suggesting that the reversibility of the first reaction does not change the long term dynamics of the reaction in the qualitative manner. However, the reversibility does affect the efficiency of the completion of reaction in the quantitative manner because the reverse reaction lowers the production of species z. From the dynamical point of view, system (1) is analogous to the following reaction system

$$x + y \xrightarrow{\alpha'} z, \qquad z + u \xrightarrow{\gamma'} v, \tag{2}$$

where $\alpha' \sim \alpha$ and $\gamma' \sim \gamma$ as $\beta \ll \alpha$.

In the model, we assume that the fragments rejoining is an irreversible process. In other words, after two pieces of fragments join together, the resulting fragment does not split. Thus the combination of recruitment process and rejoining process forms a system similar to (1) if the recruitment is reversible, while the irreversible approximation of recruitment process leads to system (2). Therefore, we conclude that the reversibility of the recruitment process may change the biphasic profile of the mean rejoining time slightly in the quantitative manner, but not in the qualitative manner.

Supplementary Note 2: System of Biochemical Reactions

The DNA fragments rejoining involves three steps of reactions and leads to a large system of biochemical reactions. To study the model, we need to identify all the involving species and reactions.

Biochemical Reactions

Taking into consideration all the reactions involving in the three steps of DNA fragments rejoining, we have a series of biochemical reactions listed below

$$\begin{array}{ll}
X_n + E \xrightarrow{k_1} X_n^E, & n \geq L_m, \\
X_n^E + E \xrightarrow{k_1} X_n^{EE}, & n > L^*, \\
X_n^E + X_m^E \xrightarrow{k_2} X_{n+m}^R, & L_m \leq n, m \leq L^* \\
X_n^E + X_m^{E*} \xrightarrow{k_2} X_{n+m}^{r*}, & L_m \leq n \leq L^* < m, \\
X_n^{E*} + X_m^{E\star} \xrightarrow{k_2} X_{n+m}^{*\star}, & n, m > L^*, \\
X_n^R \xrightarrow{k_3} X_n, & \\
X_n^{r*} \xrightarrow{k_3} X_n^*. &
\end{array} \tag{3}$$

where $*$ and $\star$ are repair protein E, protein residue r or none $\emptyset$. Note that some species can be produced through different reactions. For instance,

$$\begin{array}{rcl}
X_n^E + X_m^E & \xrightarrow{k_2} & X_{n+m}, \\
X_{n-1}^E + X_{m+1}^E & \xrightarrow{k_2} & X_{n+m}, \\
X_{n+m}^r & \xrightarrow{k_3} & X_{n+m},
\end{array}$$

as $n, m > L^*$.

Reacting Species

Assume that DNA fragments of different length are different species. When the DNA fragments of the same length are treated to be distinct with or without repair protein E, the protein residue r or R attached, more species will be introduced into the system. Collectively, all the species involving in the biochemical reactions of DNA fragment rejoining are listed below

$$\mathbb{X} = \big(X_n, X_n^E, X_n^{EE}, X_n^{rE}, X_n^r, X_n^{rr}, X_n^R\big). \tag{4}$$

Because extremely short fragments ($n < L_m$) cannot recruit Ku, X_n^E exists only if $n \geq L_m$ and similarly X_n^{EE} exists for $n > L^*$. By considering how other species are produced, we may notice that X_n^R is well defined for $2L_m \leq n \leq 2L^*$, X_n^r and X_n^{rE} for $n > L_m + L^*$ and X_n^{rr} for $n > 2L^*$. Because of these seven types of derivative species with the same length, a large amount of species have to be included into the system when different lengths are taken into consideration.

Large Network of Reactions

If we let $\#\big(X_n\big)$ be the number of DNA fragments of length n and assume that the total length of a DNA sequence is L_T, then

$$L_T = \sum_{\substack{1\leq n\leq L_T \\ *,\star\in\{E,r,R,\emptyset\}}} n\#\big(X_n^{*\star}\big),$$

whenever $X_n^{*\star}$ is well defined, where $\#\left(X_n^{*\star}\right)$ may be zero. As L_T is large, the DNA fragments rejoining leads to a large network system consisting of approximately $6L_T$ species and $\frac{9}{16}L_T^2$ reactions. Note that short fragments of length $n < L_m$ do not participate in any reaction.

Supplementary Note 3: Stochastic Simulation by Gillespie's Algorithm

Simulation of such a large system is computationally expensive, especially when stochastic simulation is adopted. It is known that 1Gy ionizing radiation produces $25-35$ DSBs [1] and hence similar amount of DNA fragments. Therefore, the computational cost can be tremendously reduced if we consider only the possible involving reactions associated with the prescribed initial fragments distribution, instead of all the reactions.

Gillespie's Algorithm

Precisely, for a given positive integer K_0, let $\{(n_k^0, m_k^0)\}$ be a sequence of positive integer pairs such that $n_k^0, m_k^0 \geq 1$ for $1 \leq k \leq K_0$. Then a prescribed initial DNA fragments distribution can be written as

$$\mathbb{S}_0 = \{(X_{n_k^0}, m_k^0),\ k = 1, 2, \ldots, K_0\},$$

which consists of m_k^0 pieces of DNA fragments of length n_k^0. Denote by $\mathbb{R}$ the set of all possible reactions associated to a given set $\mathbb{S}$ of species, then it is readily to see that

$$\mathbb{R}_0 = \left\{X_{n_k^0} + E \xrightarrow{k_1} X_{n_k^0}^E,\ \text{ if } \ n_k^0 \geq L_m\right\}_{k=1,\ldots,K_0}.$$

with the associated propensity functions defined by

$$a_k^0 = \frac{k_1}{V} X_{n_k^0} E, \qquad \text{if} \quad n_k^0 \geq L_m.$$

Set

$$a_0^0 = \sum_{1\leq k\leq K_0, n_k^0\geq L_m} a_k^0.$$

By the Gillespie's direct algorithm of stochastic simulation [2], pick randomly a pair of real numbers (r_1^0, r_2^0) satisfying uniform distribution on the interval $[0, 1]$. Then the first reaction time is given by

$$\tau_1 = \frac{1}{a_0^0} \ln\left(\frac{1}{r_1^0}\right), \tag{5}$$

and the first firing reaction is reaction j_1 satisfying

$$\sum_{1\le k\le j_1, n_k^0 \ge L_m} a_k^0 > r_2^0 a_0^0 \ge \sum_{1\le k\le j_1-1, n_k^0 \ge L_m} a_k^0. \tag{6}$$

Consequently at time $t = \tau_1$ when the first reaction step is completed, the set of reacting species is updated to

$$\mathbb{S}_1 = \left\{ \left(X_{n_k^0}, m_k^0\right),\ k \ne j_1;\ \ \left(X_{n_{j_1}^0}, m_{j_1}^0 - 1\right), \left(X_{n_{j_1}^0}^E, 1\right) \right\}.$$

Hence the associated reaction set $\mathbb{R}_1$ and propensity functions $\{a_k^1\}$ are revised correspondingly. Repeat this process of Gillespie's stochastic simulation until the reaction set $\mathbb{R}$ is an empty set, that is, no more reactions can happen. Note that the propensity functions of the second order reactions for fragments rejoining are given by

$$\frac{k_2}{V} X_n^{E*} X_m^{E\star} \quad \text{or} \quad \frac{k_2}{2V} X_n^{E*}\left(X_n^{E*} - 1\right)$$

as X_n^{E*} and $X_m^{E\star}$ are different or the same species, respectively. In the release step that is a first order reaction, the propensity functions are of the forms

$$k_3 X_n^{r*} \quad \text{or} \quad k_3 X_n^R.$$

As a summary, the Gillespie's stochastic simulation is preformed in the following steps.

0. Initialize DNA fragments distribution $\mathbb{S}_0$ and reaction set $\mathbb{R}_0$ at $t = 0$.
1. Calculate propensity function a_0 according to $\mathbb{R}$.
2. Choose (r_1, r_2) satisfying uniform distribution on $[0, 1]$.
3. Calculate τ and j as in (5) and (6), respectively.
4. Update the set of reacting species $\mathbb{S}$ and reaction set $\mathbb{R}$ at $t = t + \tau$.
5. End simulation if $\mathbb{R}$ is empty or return to step 1 otherwise.

Kinetics of DNA Fragments Rejoining

To mimic the kinetics of DNA fragment rejoining, we need to record the number or the fraction of DNA fragments in each step of reactions. In other words, we count

$$M_T(t_i) = \sum_{\substack{1\le k\le K_i \\ *,\star\in\{E,r,R,\emptyset\}}} \#\left(X_{n_k^i}^{*\star}\right)$$

after reaction step i is completed, where $\#(X_{n_k})$ is the number of fragments of length n_k. Because DNA fragments rejoining is assumed to be irreversible, its kinetic profile must be in the non-increasing manner and undergo no change after all the repairable fragments are rejoined.

Moreover, due to the stochastic features of the DNA fragment rejoining, the kinetic changes and total rejoining time may be very different although the simulations start from the same initial fragments distribution and share the same decreasing profile. Therefore, the mean value of total rejoining times obtained by numerous simulations is considered as the mean rejoining time associated to a specific initial fragments distribution, as used in Figures 3(a-d).

Supplementary Note 4: Comparison with Experimental Data

Parameters

Ku is an abundant heterodimeric nuclear protein (Ku70/Ku80). It was estimated that around half a million copies of Ku70/80 complex are produced in the human cells [3]. Hence, as suggested in [4] that there is an optimal level of repair enzymes within cells for optimizing DNA repair and the precise value of the repair enzyme concentration is of some relevance, we suppose, in this model, that the amount of repair protein E remains constant in a relative normal human cell line and

$$E = 4 \times 10^5.$$

Because the recruitment of repair proteins and fragments rejoining are all second order reactions, the reaction rate will be dependent on the volume of the reacting solution. Since DNA is located in the nucleus and all the reactions take place in the nucleus, we take the volume V of the nucleus for this purpose and assume that

$$V = 2 \times 10^{-10}\mathsf{ml},$$

where ml is milliliter. It is known that Ku starts accumulation at DSBs within a few seconds and reaches its maximum at around 3 minutes [5]. Thus we assume this rate is given by $\bar{k}_1 = 10/\mathsf{minute} = 600/\mathsf{h}$, where h is hour. Because $\bar{k}_1$ depends on the concentration of Ku and the volume V, we have

$$\bar{k}_1 = k_1 \frac{E}{V} \quad \Longrightarrow \quad k_1 = 3 \times 10^{-13}\mathsf{ml/h}.$$

Recent study revealed that Ku releasing from DNA is through ubiquitylation and dependent on the length of DNA. Effective removal of Ku from DNA requires DNA longer than 50bp and may take 30-60 minutes [6]. Then we assume that the repair protein residue is released in the similar time range and set

$$k_3 = 1/\mathsf{h}.$$

Up to date, there is no published data for measuring or estimating the second order reaction rate constant k_2, which is related to the fragments rejoining rate $\frac{k_2}{V} X_n^{E*} X_m^{E\star}$. Alternately, DNA ligation rates were proposed and set to be 0.03 in the fast repair kinetics and 0.003 in the slow kinetics [7] (Supplementary Document: Table S2). Thereby, we set

$$\bar{k}_2 = \frac{k_2}{V} = 0.02/\mathsf{h} \quad \Longrightarrow \quad k_2 = 4 \times 10^{-12}\mathsf{ml/h}.$$

Comparison with Experimental Data

The kinetic data of DSBs repair is achieved by taking the average value of fractions of foci observed in the experiments. In order to compare with experimental data, we need the average kinetics of fragment rejoining. Assume that there are N sets of kinetic data by N simulations,

$$\left\{ \left(t_i^j, M_T(t_i^j) \right), i = 1, \ldots, P_j \right\}, \qquad j = 1, \ldots, N,$$

where $\{t_i^j\}_{i=1}^{P_j}$ is a set of P_j time points that is either regular (equally spaced) or irregular (not equally spaced) and any two sets of time points may have no overlap. Because we know that each kinetic profile is non-increasing, we can choose N decreasing functions $f_j(t)$, each of which interpolates one set of kinetic data and is well defined at the given P time points, say $\{\bar{t}_i\}_{i=1}^{P}$, where we like to compare with experimental data. Hence we take the averaged function

$$\bar{f}(t) = \frac{1}{N} \sum_{i=1}^{N} f_i(t)$$

to describe the averaged kinetics from simulation data. Finally, the comparison is conducted between the experimental data set and interpolated simulation data set $\{\bar{f}(\bar{t}_i)\}$, as seen in Figure 4.

On the other hand, in the biological experiments, DNA double strand breaks (DSBs) are marked by 53BP1 foci. DSBs repair is testified by counting the numbers of foci at specific time points to recover the repair kinetics [8]. Complete DSBs repair results in zero foci present. In contrast, our model is for DNA fragments rejoining, whose kinetics shows the change of number of fragments and that complete rejoining leads to one fragment at last. To compare with experimental data, therefore, we consider the kinetic change of the ratio

$$\frac{M_T(t) - 1}{M_T(0) - 1}$$

which represents the ratio of DSBs and hence foci remaining. Here $M_T(t)$ is the total number of DNA fragments and $M_T(t) - 1$ the total number of DSBs at time t.

References

1. Gulston M, Fulford J, Jenner T, de Lara C, O'Neill P (2002) Clustered DNA damage induced by γ-radiation in human fibroblasts (HF19), hamster (V79-4) cells and plasmid DNA is revealed as Fpg and Nth sensitive sites. Nucleic Acid Res. 30(15): 3464-3472 .

2. Gillespie DT (2007) Stochastic simulation of chemical kinetics. Annual Rev Phys Chem, 58: 35-55.

3. Anderson CW, Carter TH (1996) The DNA-activated protein kinase – DNA-PK. Curr Top Microbiol Immunol. 217: 91-111.

4. Richard M, Fryett M, Miller S, Booth I, Grebogi C, et al. (2011) Optimality in DNA repair. J Theor Biol. 292:39-43.

5. Mari PO, Florea BI, Persengiev SP, Verkaik NS, Brüggenwirth HT, et al. (2006) Dynamic assembly of end-joining complexes requires interaction between Ku70/80 and XRCC4. Proc Natl Acad Sci USA, 103(49): 18597-18602.

6. Postow L, Ghenoiu C, Woo EM, Krutchinsky AN, Chait BT, et al. (2008) Ku80 removal from DNA through double strand break-induced ubiquitylation. J Cell Biol. 182(3): 467-479.

7. Zhang XP, Liu F, Cheng Z, Wang W (2009) Cell fate decision mediated by p53 pulses. Proc Natl Acad Sci USA, 106(30): 12245-12250.

8. Asaithamby A, Hu B, Delgado O, Ding L-H, Story MD, et al. (2011) Irreparable complex DNA double-strand breaks induce chromosome breakage in organotypic three-dimensional human lung epithelial cell culture. Nucleic Acids Res, 39 (13): 5474–5488.
